# Molecular evidence of high rates of asymptomatic *P. vivax* infection and very low *P. falciparum* malaria in Botswana

**DOI:** 10.1186/s12879-016-1857-8

**Published:** 2016-09-29

**Authors:** Thato Motshoge, Grace K. Ababio, Larysa Aleksenko, John Read, Elias Peloewetse, Mazhani Loeto, Tjantilili Mosweunyane, Kentse Moakofhi, Davies S. Ntebele, Simon Chihanga, Mpho Motlaleng, Anderson Chinorumba, Moses Vurayai, Jeffrey M. Pernica, Giacomo M. Paganotti, Isaac K. Quaye

**Affiliations:** 1 Ministry of Health, Gaborone, Botswana; 2 University of Ghana School of Allied Health and Biomedical Sciences, Accra, Ghana; 3 Department of Pathology, University of Namibia School of Medicine, Windhoek, Namibia; 4 School of Medicine, University of Botswana, Gaborone, Botswana; 5 Biological Sciences Department, University of Botswana, Gaborone, Botswana; 6 Ministry of Health, National Malaria Control Program, Gaborone, Botswana; 7 World Health Organization, Botswana Country Office, Gaborone, Botswana; 8 Division of Infectious Disease, Department of Pediatrics, McMaster University, Hamilton, ON Canada; 9 University of Botswana-University of Pennsylvania Partnership, Gaborone, Botswana; 10 Department of Medicine, Perelman School of Medicine, University of Pennsylvania, Philadelphia, PA USA; 11 University of Namibia School of Medicine, Windhoek, PBAG13301 Namibia

**Keywords:** Asymptomatic *Plasmodium vivax*, Botswana

## Abstract

**Background:**

Botswana is one of eight SADC countries targeting malaria elimination by 2018. Through spirited upscaling of control activities and passive surveillance, significant reductions in case incidence of *Plasmodium f*alciparum (0.96 – 0.01) was achieved between 2008 and 2012. As part of the elimination campaign, active detection of asymptomatic *Plasmodium* species by a highly sensitive method was deemed necessary. This study was carried out to determine asymptomatic *Plasmodium* species carriage by nested PCR in the country, in 2012.

**Method:**

A cross-sectional study involving 3924 apparently healthy participants were screened for *Plasmodium* species in 14 districts (5 endemic: Okavango, Ngami, Tutume, Boteti and Bobirwa; and 9 epidemic: North East, Francistown, Serowe-Palapye, Ghanzi, Kweneng West, Kweneng East, Kgatleng, South East, and Good Hope). Venous blood was taken from each participant for a nested PCR detection of *Plasmodium* species.

**Results:**

The parasite rates of asymptomatic *Plasmodium* species detected were as follows: *Plasmodium falciparum*, 0.16 %; *Plasmodium vivax*, 4.66 %; *Plasmodium malariae*, (*Pm*) 0.16 %; *Plasmodium ovale*, 0 %, mixed infections (*P. falciparum* and *P. vivax*), 0.055 %; and (*P. vivax* and *P. malariae*), 0.027 %, (total: 5.062 %). The high proportion of asymptomatic reservoir of *P. vivax* was clustered in the East, South Eastern and Central districts of the country. There appeared to be a correlation between the occurrence of *P. malariae* infection with *P. vivax* infection, with the former only occurring in districts that had substantial *P. vivax* circulation. The median age among 2–12 year olds for *P. vivax* infection was 5 years (Mean 5.13 years, interquartile range 3–7 years). The odds of being infected with *P. vivax* decreased by 7 % for each year increase in age (OR 0.93, 95 % CI 0.87–1.00, *p* = 0.056).

**Conclusion:**

We have confirmed low parasite rate of asymptomatic *Plasmodium* species in Botswana, with the exception of *P.vivax* which was unexpectedly high. This has implication for the elimination campaign so a follow up study is warranted to inform decisions on new strategies that take this evidence into account in the elimination campaign.

## Background

Malaria disease still kills close to half a million people annually with 90 % of the deaths occurring in sub-Saharan Africa [1]. Recently the World Health Assembly endorsed a new Global Technical Strategy for malaria 2016–2030, which included reducing the case incidence and mortality of malaria by 90 % and target elimination in 35 countries [2]. Botswana is one of eight Southern Africa Developing Countries (SADC) targeting malaria elimination by 2018, [3] after achieving significant reduction in case incidence (0.97-0.01) from 2008 to 2012 [4, 5], attributed to a spirited upscaling of interventional activities. Malaria control activities in the country began in the 1950s with a focus on vector control. A change in focus from 2008 which included passive surveillance through monthly reports and integrated disease surveillance and reports, enabled attainment of targets for the adoption of malaria elimination strategy in 2009 (Botswana Ministry of Health, MIS, 2012, WHO world malaria report).

**Fig. 1:**
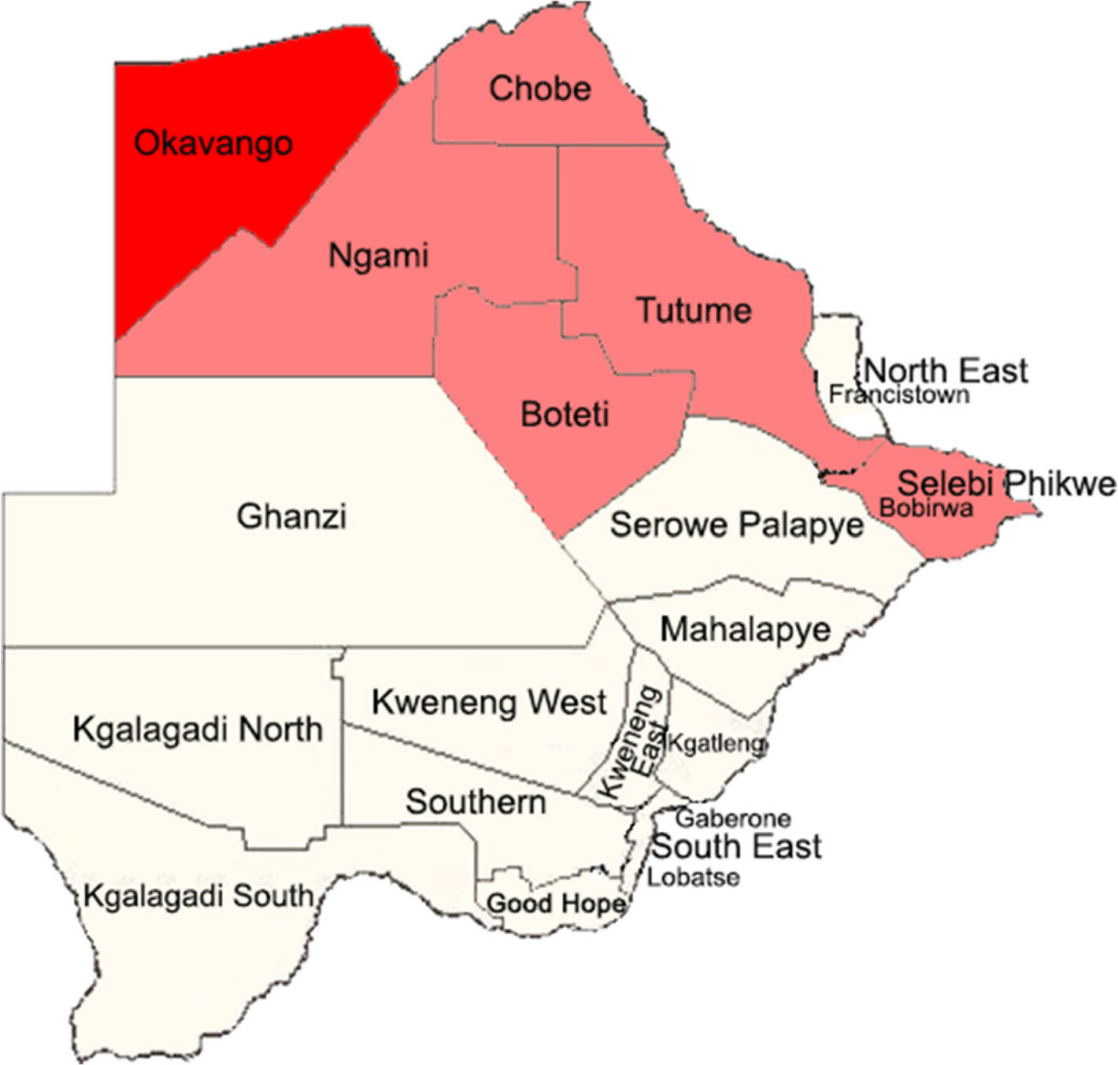
Map of malaria endemicity in Botswana). The Map was obtained from the Botswana NMCP

In general the trend in transmission in Botswana is seasonal and unstable ranging from high endemicity in the northern part of the country to very low in the southern part of the country (Fig. 1). This largely, depends on rainfall patterns and temperature [4, 6]. The northern part experiences relatively high rainfall in the summer and relatively mild winter temperatures. In contrast, the southern part of the country experiences relatively low rainfall in the summer and relatively low winter temperatures. Out of the five different species of the genus *Plasmodium* known to infect humans, (*Plasmodium falciparum*: *Pf*, *Plasmodium vivax*: *Pv*, *Plasmodium ovale*, *Plasmodium malariae* and *Plasmodium knowlesi*) *Pf* infection is the major known cause in Botswana. It is estimated that 98 % of malaria infections are *Pf*, while 2 % is attributed to *Pv* [4]. The human parasite reservoir is made up of symptomatic and asymptomatic individuals. The latter is recognized to constitute a significant proportion of the parasite reservoir and serve as a significant source of transmission [7–9] as the individuals do not seek health care. For an elimination strategy to be successful, it is crucial that asymptomatic and submicroscopic reservoirs of *Plasmodium* species are sought out actively for targeted intervention. It is also well established that in malaria elimination countries, as *Pf* incidence rate goes down *Pv* incidence rate increases [10], because of the unique biological characteristics of the parasite. These include dormant liver stage that results in relapses even after treatment, development in mosquito vectors at lower ambient temperatures and therefore has a large span of ecologically induced infectivity, and its lower parasite density that limits detection microscopically and by RDT. Clearly for an elimination strategy, highly sensitive methods of detection are also required [11, 12]. As part of the malaria elimination strategy in Botswana, the first active surveillance of asymptomatic *Plasmodium* spp was undertaken in 2012. This was carried out throughout all defined zones of endemicity in the country, using PCR methodology for the case detection. The choice of PCR methodology was informed by the fact that it is well recognized that PCR consistently detects at least twice as many infections as light microscopy and therefore an excellent choice when high sensitivity is desired [13, 14]. The work was undertaken to provide guidance in interventional activities towards focal areas of transmission especially for asymptomatic individuals, so that informed decisions on new strategies towards the malaria elimination agenda are made. The present article presents the findings.

## Method

### Study Site

Study sites were selected with the support of Statistics Botswana from five endemic and nine non-endemic districts in Botswana. These were respectively, Okavango, Ngami, Tutume, Boteti and Bobirwa (Endemic areas), and North East, Francistown, Serowe-Palapye, Ghanzi, Kweneng West, Kweneng East, Kgatleng, South East, and Good Hope (epidemic areas) (Fig. 1). Within each district, the Ministry of education primary school register and Ministry of Health Clinic/Health post registers were used to randomly select the schools and clinics from which enrolment was done.

### Study subjects and blood sample collection

The study included 3624 subjects of ages 2–12 enrolled within a month in May 2012, in the identified catchment area schools, welfare clinics and healthposts, using a multistage sampling strategy. The first stage was a purposeful selection of districts with known malaria transmission, classified as either highly endemic, moderate, low or sporadic, based on recommendations from the National Malaria Control Program (NMCP). In the second stage which was also purposeful, towns within the districts that constituted the focal areas of transmission were selected based on NMCP recommendation, using guidelines on classification of malaria transmission endemicity. This was to ensure that areas of high, moderate, low and sporadic transmissions with distinct characteristics in each community were captured in the sampling process. In the third stage, schools/clinics within each town were purposefully selected using a two stage clustering approach based on population density and adequate cross sectional representation of the communities to avoid any bias. In the final stage, participants were assigned numbers and randomly enrolled following informed parental consent and consent of heads of schools and clinics. The total number of samples obtained per district/town was directly proportional to the estimated population. The formula used in calculating the sample size was: *n* = (*z*^*2*^)(*r*)(*1*-*r*)(*f*)(*k*)/(*p*)(*ñ*)(*e*^*2*^). Using an estimated *Pv* known prevalence for Africa (*r*) of 10 %); *z* statistic of 1.96 for the 95 % Confidence Interval, design effect (*f*) of 2.4; non response rate (*k*) of 8 %; the proportion of the population under 12 (*p*) of 0.34; average household size (*ñ*)of 3.4; and margin of error (*e*) of 8 %; a sample size of 897 households was obtained from which the total number of samples were derived based on the population of the districts. Children aged 6–12 years were largely from the primary schools while those aged from 2 to 5 were enrolled from the child welfare clinics and health posts. The subjects from the clinics did not present with any disease except normal growth monitoring follow up visits. All the enrolled children were asymptomatic for malaria. Asymptomatic subjects were individuals with body temperatures <37.5 °C at the time of sample collection and without a history of fever in the preceding 72 h. A subject was enrolled if the individual had no complaints related to malaria, had not left the village for a year at least and parental consent was given for blood to be taken. The sample collection was timed to coincide with the onset and peak of the malaria season in the country.

After consent from the guardian or parent of the subjects, a short clinical assessment of tympanic temperature was done and history of malaria and travel requested. Subsequently, basic demographic information on age and sex was documented. A 2.5 to 3 ml venous blood was collected into EDTA vacutainer tubes from which three Dried Blood Spots (DBS) were made unto whatman number 3 filter papers enclosed in zip lock bags with desiccators each. Samples were stored in ice packs (DBS papers were retained at room temperature) and transported to the laboratory within 30 min. Blood was separated by centrifugation into plasma and cell pellets which were frozen at -20 °C and subsequently -80 °C till analyzed.

### Laboratory analysis

#### DNA extraction

Genomic DNA (gDNA) was isolated from red blood cell pellets (100 μl, corresponding to 200 μl of whole blood) using the QiaAmp DNA Blood mini kit (Qiagen Inc., Valencia, CA) or Quick-gDNA™ Blood Mini Prep (Zymo Research, USA) and quantified with the Fisher Thermo Scientific Nanodrop 2000/2000C USA. All DNA were eluted into 100 μl elution buffer and stored at -80 °C till used for PCR.

#### Molecular detection of *Plasmodium* species

All *Plasmodium* species were detected by a slight modification (with respect to the amount of DNA used in the reactions, total volume of PCR reactions, type and quantity of DNA polymerase used and cycling conditions) of the double nested PCR procedure targeting conserved species specific regions of the small subunit 18S ribosomal RNA (18S ssRNA) [15]. The first nested reaction was genus specific, and if positive, was followed by a species specific nested reaction. All detection assays were single-plexed and run in a 96 well plate Roche light cycler platform. The PCR kits were the Qiagen PCR core kit (Qiagen Inc, Valencia, CA). Species specific primers were ordered from Eurogentec, Liege, Belgium and control *Plasmodium* species DNA, from ATCC, Virginia, USA. The primer sequences were exactly as published previously [15].

All amplification reactions were carried out in a total volume of 25 μl and in the presence of 10 mM Tris-HCl, pH 8.3, 4 mM MgCl_2_, 50 mM KCl, 250nM of each oligonucleotide primer, 200 μM of each of the four dNTPs and 0.5 units of Dream Taq DNA polymerase. 5 μl of DNA prepared from whole blood was used to initiate the genus specific primary amplification PCR reaction and 2 μl of the product was then used in the secondary amplification including the species specific detection assays. The PCR cycling parameters for the primary amplification were as follows: initial denaturation at 94 °C for 10mins, preceded the cyclin conditions which consisted of denaturation at 94 °C for 30 s, annealing at 55 °C for 1 min and extension at 72 °C for 1 min for 35 cycles. After the final annealing step followed by extension at 72 °C for four minutes, the reaction was ended. In the second nested PCR, annealing temperature of 62 °C was used. For the species specific amplifications the only changes were initial denaturation at 94 °C for four mins and annealing at 58 °C.

To ensure that there was no cross contamination, negative control samples (no DNA template) were randomly included in the run. The consistency of the results was checked by multiple runs of a number of subsets of the samples which were randomly picked.

The amplified products were analyzed on 2 % agarose gels by electrophoresis, followed by visualization on a UV transilluminator after ethidium bromide staining.

#### Statistical analysis

Data were entered into an Excel data sheet and used in SPSS and STATA v11.2 (StataCorp, College Station, TX, USA) for analysis. Descriptive statistics were provided for relevant demographic covariates. Comparisons of categorical variables were done with chi-square testing or Fisher’s exact test. Factors influencing the likelihood of malaria infection were explored using forward stepwise logistic regression.

## Results

### Study population

A total of 3624 children across 14 districts were assessed for *Plasmodium* species. The mean age of all enrolled subjects was 5.48, which was distributed as follows: <2 years, 0.084 %; 2- < 5, 41 %; 5- < 9, 47.27 %; >9 years, 11.65 %; while gender was 51.1 % female. None of the participants had traveled outside the country for the past year.

### Prevalence of Plasmodium species

**Fig. 2:**
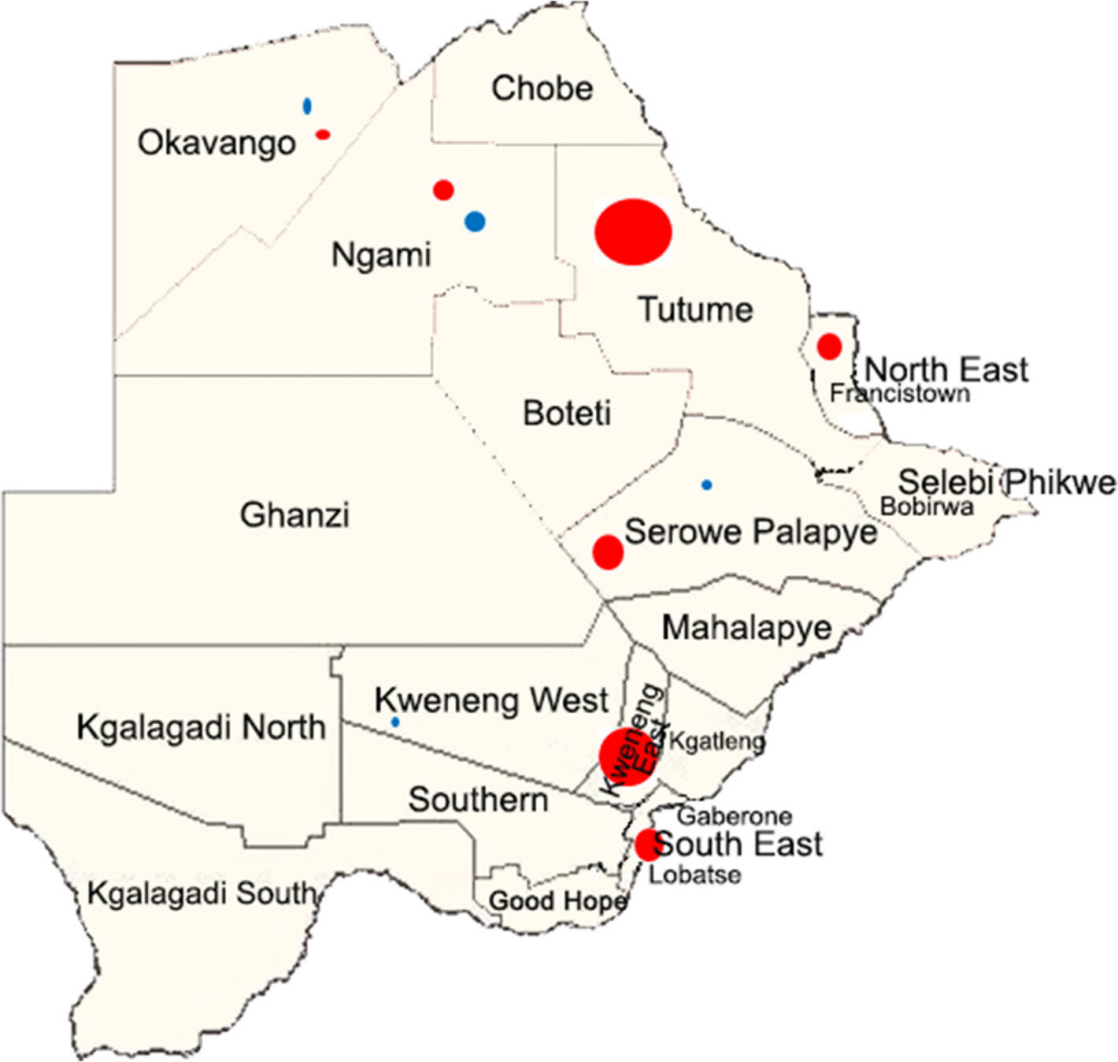
District map of Botswana showing areas of *Pv* () and *Pf* () infections. The size of a circle is proportional to the prevalence based on the total positives/total samples tested for the respective district. (The unannotated Map was obtained from the Botswana NMCP)

Overall 4.99 % of individuals had *Plasmodium* species infection segregated as follows: *Pf*, 0.16 %; *Pv*, 4.66 %; *P. malariae* (*Pm*), 0.16 %; *P. ovale*, 0 %, mixed infections (*Pf* and *Pv*), 0.055 %; and (*Pv* and *Pm*), 0.027 %. *Pv* infection accounted for 93.37 % of the *Plasmodium* species infection in the population. The distribution of *Plasmodium* species infection by district is shown in Table 1. The *Pv* infection was clustered around East (Tutume, Francistown), South East (Serowe-Palapye and South East) and Central districts (Kweneng East) (Fig. 2). These areas of *Pv* infections do not correlate geographically with *Pf* infections in the established zones of endemicity, however they overlap partially with newly identified hotspots in the country. We noted from the NMCP that the indicated areas have a higher proportion of migrants who may carry latent infections. The odds that a child resident would be infected with *Pv* species was 9.19 times higher (95 % CI 5.50–15.4, *p* < 0.001) in the Tutume region and 7.10 times higher (95 % CI 4.41–11.4, *p* < 0.001) in the Kweneng East region than in the rest of the districts that had detectable *Pv* infection. The median age of those infected with *Pv* was 5 years (Mean 5.13 years, interquartile range 3–7 years) while those not infected with any species of *Plasmodium* had a median age of 6 years (mean 5.50 years, Interquartile range 3–8 years). The age breakdown for those infected with *Pv* was: <5 years, 72 positives from 1466 specimens (4.91 %); 5- < 9 years, 65 positives from 1688 specimens (3.85 %); 9–12 years, 16 positives from 416 specimens (3.85 %). These differences in positivity between age categories were not statistically significant. However, the odds of being infected with *Pv* decreased by 7 % for each year increase in age (OR 0.93, 95 % CI 0.87–1.00, *p* = 0.056). Districts with higher numbers of *Pv* infections appeared to have higher numbers of *Pm* infections as well; *Pm* infections were only detected in the district with the most *Pv* transmissions (Kweneng East), the second-most *Pv* infections (Tutume), and the Francistown district. We did not encounter any problem with contamination of samples.

**Table 1:** The distribution of asymptomatic *Plasmodium* species infection by district

District(n)	Plasmodium species		
	*P. falciparum* (*Pf*)	*P. vivax* (*Pv*)	*P. malariae* (*Pm*)
Okavango(208)	1	1	0
Ngami(80)	1	1	0
Tutume(320)	0	54	2
Boteti(181)	0	0	0
Bobirwa(203)	0	0	0
North East(160)	0	0	0
Francistown(195)	0	4	1
Serowe-Palapye(361)	1	11	0
Ghanzi(163)	0	0	0
Kweneng West(375)	1	0	0
Kweneng East(687)	0	93	3
Kgatleng(358)	0	0	0
South East(174)	0	5	0
Good Hope(159)	0	0	0
Total (3624)	4	169^a^	6

^a^The odds of being infected with *Pv* decreased by 7 % for each year increase in age (OR 0.93, 95%CI 0.87–1.00, *p* = 0.056)

## Discussion

The Botswana malaria elimination program has clearly been on track as the asymptomatic *Pf* reservoir determined in this study is only 0.16 %. However, our identification for the first time of a significant asymptomatic *Pv* reservoir in the country has brought in a new paradigm for the elimination program that requires immediate attention, in order to sustain the gains made to date and achieve the new target of elimination by 2018. Our findings add to the growing evidence of the presence of *Pv* in Africa. As has been observed in other countries, the reservoir of *Pv* was clustered, [16, 17] centering in Tutume and Kweneng East as major hotspots with minor hotspots to the East and South Eastern districts. It is not clear to us at the moment why the infection was clustered in the identified hotspots, except that the communities have a “rural” setting as have been observed elsewhere [16]. In addition we learnt from the NMCP that a significant number of the inhabitants in these areas are migrant workers so it is probable that in some time past they may have travelled and harbored hypnozoites. Liu et al [18] recently suggested that in populations of high Duffy negativity, one probable source of infection will be zoonotic, with the primary source being ape reservoirs. The transmission between apes and humans is reported to be facilitated by *An. moucheti* and *An. vinckei* [18–20]. We observed that the age of the population group at risk was localized largely within the 5–7 year olds, with the odds decreasing with increasing age. This is in consonance with similar observations in Ethiopia and Peru [17, 21]. It has been argued that the population group that has the highest probable exposure to competent vectors is at the most risk of infection. In this regard a recent article from Botswana covering the period from 2012 to 2014, reported that the age group greater than 14 years had the highest number of malaria cases detected by RDT [22]. It appears that the rigorous indoor spraying and education on the use of insecticide treated bed nets by the NMCP, is impacting on indoor feeding of vectors and causing a shift in the vulnerable population group in the country. There is an evident need for further interrogation of factors that drive *Pv* transmission within specified populations in Botswana and sub-Saharan Africa.

The role of *Pv* in malaria transmission and elimination in sub-Saharan Africa is increasingly drawing attention. Infection of rbcs by *Pv* is traditionally established to be attributed to positivity for the Duffy antigen, also called Duffy antigen receptor for chemokines (DARC) [23–25]. A significant number of the population in Africa has a null phenotype so that transmission of *Pv* is considered an unlikely scenario. However, past and recent reports from several countries in sub-Saharan Africa, including Liberia, Senegal, Equitorial Guinea, Angola, Ethiopia, Cameroon, Kenya and Malagasy, have shown that Duffy negativity alone does not preclude infection with *Pv* [26–31]. The Duffy negativity rate in Southern Africa including Botswana is estimated between 0 and 30 % [24], so the findings are of particular interest, regarding the Duffy positivity rate in the affected population. It may also be that some environmental factor is driving the infection of Duffy null erythrocytes through a yet to be identified receptor. It is interesting that from a recent map of Duffy negativity in Botswana, the most negative population is in the Eastern sector where the cluster of *Pv* infections were found [16]. Further efforts at probing into potential mechanisms of *Pv* infection, may first be focused on host characteristics within the populations including the environment, parasite genetic variability and available competent vectors. Recently a report on modeling of *Pv* infection dynamics revealed that up to 90–96 % of *Pv* infection in endemic areas is due to hypnozoite reactivation [32]. A major factor that has been suggested to induce reactivation is host stress factors (eg, infectious disease exposure) [33, 34]. We observed an apparent correlation between *Pm* infection and *Pv* infection in this study (though the lack of *Pm* cases precludes the demonstration of a statistically significant association) but as to whether they play complimentary roles in infectivity requires further interrogation. *Pv* appears to show high clonal diversity in endemic regions, so that genotyping approaches will also be necessary in elucidation of the infection dynamics.

Our findings also highlight the fact that active case detection (ACD) in malaria elimination is crucial in the identification of hotspots. ACD is expensive so malaria elimination programs need to think through appropriate models that are cost effective. Two suggested models are *reactive case detection* and *proactive case detection*. In a reactive case detection model, a trigger of a passive case leads to the screening of households including neighbors [35]. The limitation in this case is defining the number of neighboring households that form a critical threshold for screening. In a proactive case detection, known or recognized hotspots are screened periodically in the absence of any trigger, passively. In adopting an appropriate strategy, neighboring countries within the SADC E8 need to engage in active conversation and adopt feasible and common strategies so that baseline factors on specific models can be developed and shared.

## Conclusions

We have provided molecular evidence for the first time of *Pv* asymptomatic infection in Botswana and also affirmed that the malaria control program targeting *Pf* has indeed been on track. New and concerted strategies to achieve elimination of malaria in 2018 in Botswana are urgently required as are efforts at better understanding of characteristics of susceptible host, environmental factors and *Pv* biological characteristics.

## Abbreviations

NMCP: National Malaria Control Program
ACD: Active Case Detection
ATCC: American Type Culture Collection
MIS: Malaria Indicator Survey
Pf: *Plasmodium Falciparum*
Pm: *Plasmodium malariae*
Pv: *Plasmodium vivax*
RDT: Rapid Diagnostic Test
SADC: South African Development Countries
